# Importance of pain perception after mucogingival surgery in multiple Miller class III/RT2 gingival recessions: A randomized clinical trial

**DOI:** 10.4317/medoral.26906

**Published:** 2024-12-24

**Authors:** Aitziber Fernández-Jiménez, Ana Mª García-De-La-Fuente, Ruth Estefanía-Fresco, Irene Lafuente-Ibañez-de-Mendoza, Xabier Marichalar-Mendia, José Manuel Aguirre-Urizar, Luis Antonio Aguirre-Zorzano, Eduardo Ginestal-Gómez

**Affiliations:** 1 Research Group: GIU21/042. Department of Stomatology, Faculty of Medicine and Nursing, University of the Basque Country/Euskal Herriko Unibertsitatea (UPV/EHU), Barrio Sarriena s/n, 48940 Leioa, Bizkaia, Spain; 2 Research Group: GIU21/042. Department of Nursing I, University of the Basque Country/Euskal Herriko Unibertsitatea (UPV/EHU), Bizkaia, Spain; 3 In memorial of Professor Eduardo Ginestal Gómez

## Abstract

**Background:**

Although postoperative pain after mucogingival surgery can modify the patient's daily life, few studies have compared daily postoperative pain in mucogingival surgery considering patient characteristics. The aim of this study was to evaluate postoperative pain in 24 patients with Miller class III/RT2 multiple recessions treated with the modified VISTA (m-VISTA) versus the coronally advanced flap (CAF) with a connective tissue graft (CTG).

**Material and Methods:**

Data related to pain intensity (PI), pain duration (PD), analgesic drug intake (AI), and time of analgesic need (TAN) were collected in the “UPV/EHU pain diary”. Other data were also evaluated such as the patient’s central sensitization level, pre-surgical pain, dimensions of CTG, and postoperative incidences were included. A descriptive and analytical statistical analysis was performed.

**Results:**

PI (m-VISTA = 11.19 vs. CAF = 8.10) and PD (m-VISTA = 25.27 min. vs. CAF = 10.34 min.) were higher in the test group, being statistically significant at 2 and 8 hours. TAN (m-VISTA = 63.58 min. vs. CAF = 53.25 min.) was higher in the test group, while AI was two times higher in the control group (m-VISTA = 15 vs. CAF = 38). An association was observed between PI and both the length of the SCTG and drugs/alcohol consumption.

**Conclusions:**

Postoperative pain was greater in the group of patients treated with m-VISTA. However, these patients showed a higher number of risk factors which might have increased or modified their pain symptoms.

** Key words:**Postoperative pain, pain intensity, visual analog pain scale, gingival recessions, plastic surgery, clinical trial.

## Introduction

Patient-reported outcome measures (PROMs) cover both aesthetic and therapeutic satisfaction, and short-term postoperative events that can modify the patient’s everyday life (1-3). Assessing these factors, especially in mucogingival therapy, is currently key (2) as they determine the success of the procedure and may trigger the decision-making process as to receive a similar treatment in the future; therefore, they should be carefully analyzed (1).

Acute pain is a biological process associated with tissue damage and inflammation (4) reaches its maximum peak in the first 48 hours (5). Thus, its analysis should be performed as close as possible to the time of the intervention. In dentistry, studies on postoperative pain have mainly been conducted in third-molar surgeries at hospital centers, where recorded data are easier to gather. In periodontal plastic surgery, immediate postoperative pain has been previously analyzed at the time of suture removal and the follow-up appointments mainly through the visual analogue scale (VAS) (3,6). Only two studies (7,8) have recorded the presence of pain daily, and to date, none has introduced a “pain diary” to investigate postoperative pain within the first 24 hours after treatment.

In addition, no study has evaluated the influence that patient’s characteristics or the intra- and postoperative data might have on pain. This would be helpful to try to elucidate if the surgical procedure itself or other patient-related factors could increase the pain. To analyze the surgical pain, we designed a daily pain diary where the immediate postsurgical pain and patient-related factors were taken into consideration which has been recently published (9). Furthermore, a recent systematic review (3) demonstrates that post-operative pain or discomfort are the most prevalent PROMs after root-coverage therapy (62.22%).

PROMs analysis has previously been collected either through Likert scale-type surveys designed by the researchers or with validated scales, such as health questionnaires or VAS. However, these assessments have not considered some data of patients like gender, smoking/alcohol consumption, systemic diseases, and pre-surgical pain, which might influence the PROMs of any therapeutic procedure (10).

Considering this background, the main objective of this paper was to analyze the postoperative pain experienced by the patients after going through two different mucogingival techniques, the modified VISTA (m-VISTA) (test group, TG) (9) versus the coronally advanced flap (CAF) (control group, CG) (11), for the treatment of multiple Miller class III/RT2 gingival recessions. This was the secondary variable of a previously published randomized clinical trial (RCT) (12), designed to compare both surgical techniques in terms of root coverage. In addition, the influence that multiple characteristics associated with the patient, the intervention and the postoperative period may have on this postoperative pain was also assessed (9).

## Material and Methods

- Study design and population, inclusion and exclusion criteria

We performed a triple-blind RCT (clinicaltrials.gov/ NCT03258996) following CONSORT guidelines. This study protocol was approved by the Ethics Committee of the UPV/EHU (M10/2017/042). All patients signed an informed consent, and all study procedures were performed according to the criteria included in the Declaration of Helsinki (1975; revised in 2013). Details about the study design and the main clinical outcomes have been previously reported (12).

Inclusion criteria were: 1) >18 years old; 2) multiple (≥3) Miller class III/RT2 gingival recessions with a depth of ≥2 mm; 3) recessions treated for aesthetic reasons, recurrent inflammation, progressive recession or dentin hypersensitivity; 4) absence of active periodontal pathology; 5) full-mouth plaque (13) and bleeding (14) indices ≤15%. Exclusion criteria were: 1) smoking ≥10 cigarettes/day; 2) suffering from any systemic condition that contraindicated surgery; 3) consumption of any analgesic and/or anti-inflammatory drugs in the last 72 h prior to surgery; 4) use of any drug that reduce pain perception, such and antidepressants of anticonvulsants (except selective serotonin inhibitors), and 5) pregnant or nursing women.

- Randomization, blinding and calibration

The patients were randomized in blocks of four using statistical software (IBM SPSS® Statistics 20; IBM, Chicago, IL, USA) by a clinical monitor (AMGF). The assignments were kept hidden (AMGF) in opaque envelopes until the time of the intervention. The clinical examiner (REF) was previously calibrated, obtaining an intraclass correlation coefficient >0.75 (12).

The patient, the clinical examiner (REF) and the biostatistician (XMM) were all blinded to the surgical technique. The clinical monitor (AMGF) gave patients information about the surgical procedure on their last visit.

- Surgical techniques

**Figure 1:**
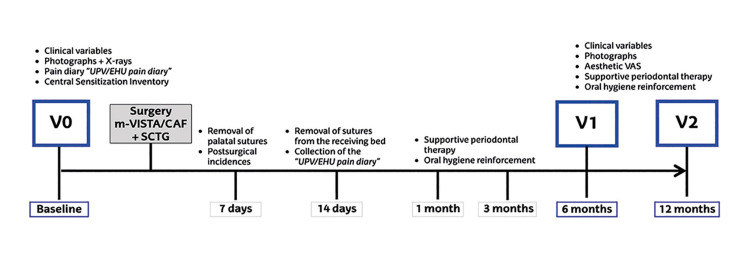
Timeline of the study.

The surgical techniques used in this study were the m-VISTA technique in the TG (9) or the CAF technique in the CG (11). All cases were treated with an autologous SCTG harvested from the palate with the UPV/EHU technique (6). Fig. 1 shows the timeline of the study (12).

- Outcome measures

All the variables were collected in the Master´s degree in Periodontology at the University of the Basque Country (UPV/EHU) (Spain). Before surgery, a blinded, experienced, and calibrated examiner (REF) recorded the periodontal clinical variables of gingival recessions (gingival recession depth, gingival recession width, probing depth, clinical attachment level, keratinized gingiva width, distance from the contact point to the interdental papilla, full-mouth plaque index, full-mouth bleeding index, and radiological bone level). Subsequently, another clinical examiner (AFJ) recorded the length, width and thickness of the SCTG obtained during the intervention; and after the intervention, another examiner (REF) recorded any immediate postoperative incidences.

The intrinsic characteristics of the patients who underwent surgery were also collected (AFJ) and all participants completed the Central Sensitization Inventory (CSI) adjusted to the Spanish population (15) in order to determine the clinical level of central sensitization (CLCS), as this could affect the postoperative pain response, independently to the intervention (16). The validated questionnaire (15) consists of two parts. Part A is a 25-item Likert-scale questionnaire where the patients answer questions related to their daily activity, choosing a single option whose score varies between: never [0), rarely [1], sometimes [2], often [3] and always [4]. The sum of all the items reveals the patient's CLCS, ranging from 0-100: subclinical [0-29], medium [30-39], moderate [40-49], severe [50-59] or extreme [60-100]. Part B is non-quantitative, and it is based on data about the disorders included in the so-called group of overlapping chronic pain conditions of central sensitization syndrome (fibromyalgia, chronic fatigue syndrome, restless legs syndrome, temporomandibular disorders and migraines or tension headache) recorded as “yes” or “no”, as well as the time of diagnosis (17).

On the day of surgery, the examiner previously recorded if the patient felt any pain in the head and neck region and/or had suffered any pain in the last month. If so, its pain intensity (PI) was assessed from 0 to 100 (0: absence of pain; 100: maximum pain experienced by the patient). After the intervention, the patient was given a "UPV/EHU pain diary" (9), designed by our research group based on the VAS. PROMs were recorded 2 and 4 hours immediately after surgery, and then every 8 hours for the first 3 days. After the first 72 hours, the patient registered the presence of postoperative pain only at night until day 7, or until complete remission of pain. Specifically, patients recorded: the greatest PI [0-100], the pain duration (PD) measured in minutes, and the need of any additional analgesic intake (AI) other than the one already prescribed (no, yes, which one?). With all of the above, mean values for PI and PD were estimated, as well as the moments at which the patient had needed AI, and the time of analgesic need (TAN), measured in minutes which was defined as the time where the patient had pain and required additional analgesia.

Finally, the presence or absence of postsurgical incidences, as well as their description, was recorded 7 and 14 days after the surgery.

- Sample size calculation

It was estimated that with an SD = 24.86% in the mean root coverage percentage (primary outcome in the original RCT (12), with an α-risk of 5% and a statistical power of 80%, 11 patients would be needed in each group. However, considering possible dropouts, the total sample was increased to 24 patients (12).

- Statistical methods

A blinded statistician (XMM) studied all variables using SPSS® Statistics 20.0 (IBM, Chicago, IL, USA), based on the patient as the unit of analysis. First, descriptive statistics were performed: means and standard deviations for quantitative variables, and frequency and percentages for categorical variables. Being a categorical variable collected only at baseline, CLCS collected through CSI (15) was treated as a quantitative variable by calculating percentages for each of the CLCS grades (subclinical, medium, moderate, severe and extreme). Non-parametric Mann-Whitney U test was then used for intergroup analytical statistics.

Finally, to analyze the possible correlation between PI and other variables, Spearman's correlation coefficient, Mann-Whitney U or Kruskal-Wallis were applied, depending on the nature of the variable. Differences were considered statistically significant when *p*<0.05.

## Results

- Study population and external validity

The CONSORT flow diagram and the clinical parameters related to root coverage have already been published (12). Baseline characteristics of the patients who underwent surgery are reported in Table 1.

A total of 24 patients were treated, 12 of which were randomly allocated to the TG [8 women; 55.26 years (SD: 7.89)] and the other 12 to the CG [6 women; 51.16 years (SD: 10.37)]. During the follow-up period, 2 patients were lost in the TG (12).

Altogether, 41.70% (n = 5) of the patients in the TG and 33.30% (n = 4) in the CG stated having experienced preoperative pain in the head and neck region in the last month (*p* = 1.00). The presence of pain on the day of surgery was reported by 16.67% (n = 2) of patients in the TG and 8.33% (n = 1) in the CG (*p* = 1.00). Thus, the mean PI during the month prior to the surgical procedure was 17.5 (SD: 23.79) and 10 (SD: 15.37) in the TG and CG (*p* = 0.55), and the mean PI on the day of surgery was 5 (SD: 11.68) and 1.67 (SD: 5.77) (*p* = 0.71), respectively.

The majority of patients showed subclinical CLCS (m-VISTA = 83.30% vs. CAF = 75.00%) and none of them had severe or extreme CLCS. Two women (16.70%) in the TG, one of them diagnosed with fibromyalgia, had moderate CLCS with presence of preoperative pain, compared to none in the CG.

When comparing the study groups at baseline, no statistically significant differences were recognized in any of the pre-surgical variables recorded.

- Intraoperative and postoperative variables

The intraoperative and postoperative variables related to pain are summarized in Table 2.

The mean length (m-VISTA = 28.88 mm vs. CAF = 26.35 mm) and width (m-VISTA = 7.44 mm vs. CAF = 6.95 mm) of the SCTG was greater in the TG; while the thickness (m-VISTA = 2.36 mm vs. CAF = 2.61 mm) was slightly greater in the CG, with the differences not being statistically significant.

**Figure 2:**
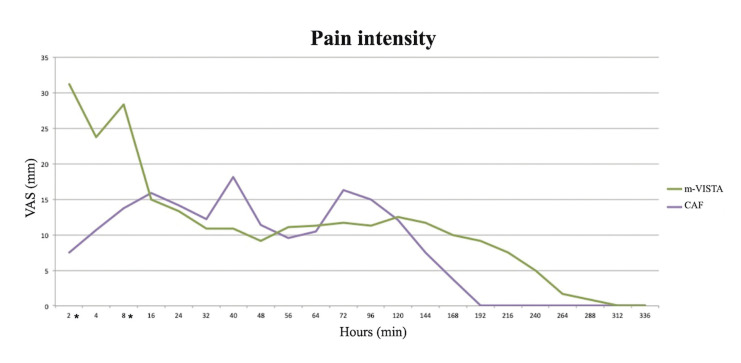
Intensity of the postoperative pain, using the “UPV/EHU pain diary”. *Statistically significant difference.

**Figure 3:**
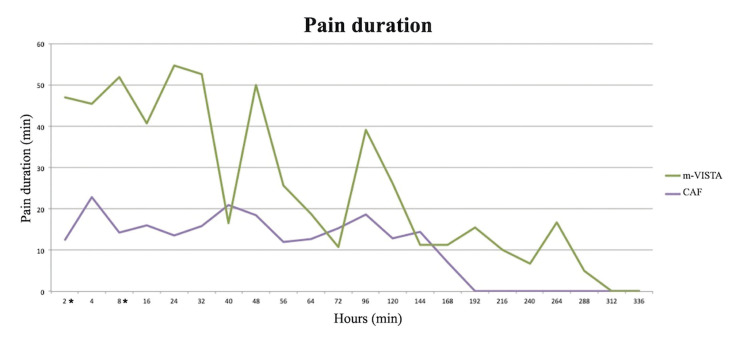
Duration of postoperative pain (minutes), using the “UPV/EHU pain diary”. *Statistically significant difference.

Regarding postoperative pain, PI (m-VISTA = 11.19 vs. CAF = 8.10) and PD (m-VISTA = 25.27 min vs. CAF = 10.34 min) were higher after the surgery in the TG, only being statistically significant at 2 hours (PI and PD; *p* = 0.001) and 8 hours (PI; *p* = 0.045 / PD; *p* = 0.010) after the surgery (Fig. 2, Fig. 3).

The TAN (m-VISTA = 63.58 min vs. CAF = 53.25 min; *p* = 0.27) was higher in the TG, with the AI being more than two times higher in the CG (m-VISTA = 15 vs. CAF = 38), but with no statistically significant differences. Two and six patients in the test and control groups, respectively, reported an additional AI at more than two recording points.

The following 12 immediate postoperative incidences were also recorded: facial hematoma (m-VISTA = 1 vs. CAF = 2), aphthae (m-VISTA = 2 vs. CAF = 1), palatal mucous necrosis (m-VISTA = 2 vs. CAF = 2), partial necrosis of the flap (m-VISTA = 0 vs. CAF = 1), partial necrosis of the graft (m-VISTA = 0 vs. CAF = 1) and postoperative bleeding (m-VISTA = 1 vs. CAF = 0).

- Postoperative pain and other variables

Table 3 summarizes all the results of the analyses carried out to explore the possible association of postoperative PI with the multiple variables that were assessed. Accordingly, a positive linear correlation was observed with the length of the SCTG, both in the CG (r = 0.619; *p* = 0.032) and in all the patients who underwent surgery, regardless of the surgical technique (r = 0.418; *p* = 0.042) (Table 3). When evaluating all the patients of the study, an association was seen between PI and drugs and/or alcohol intake (*p* = 0.023). However, no statistical association was identified between PI and patients' CLCS.

## Discussion

When analyzing the data recorded by the patients in the "UPV/EHU pain diary" (9) (PI, PD, AI and TAN), the m-VISTA technique (9), used in TG appeared to be more painful than the CAF (11) used in the CG. This result is in line with a previous study (18), where the tunneling technique caused more pain. However, it must be considered that pain experience could be influenced by both surgical and patient-related individual factors (4,19).

The overall study sample had a large number of women (14 women vs. 10 men), and the TG included two more women than the CG (m-VISTA = 66.70% vs. CAF = 50%), and a slightly higher mean age (m-VISTA = 55.26 years [41-73] vs. CAF = 51.16 years [31-63]). Women are more demanding of oral health care (20) and are more interested in aesthetics (21), which is one of the main indications for periodontal plastic surgery. However, the study groups were quite homogeneous at the beginning, and no statistically significant differences were observed in any of their general characteristics.

Regarding the intra-surgical factors of the technique-related factors, m-VISTA (9) resulted in a longer estimated chair time due to its difficulty and the locations treated within this group (mandible and posterior sectors), increasing the PI in the TG. Due to the baseline characteristics of the treated gingival recessions (12), the length of the SCTG obtained in the TG was greater than that of the CG, showing a positive linear correlation with the PI. It is well known that long intraoperative procedures promote a greater inflammatory response and, therefore, greater postoperative pain (19). Postoperative incidences were equal in number (n = 6) in both groups. However, the higher PI and PD in the TG, may have also been linked to the presence of aphthae (m-VISTA; *n* = 2 vs. CAF; *n* = 1), as in the study by Sanchez-Bernal *et al*.

Usually, studies have used AI as an indirect measure to analyze postoperative pain (3,6). However, in this study the additional AI was significantly higher in the CG, as opposed to the intensity and duration of pain experienced in this group. It is known that many patients do not take analgesics to treat pain, but to prevent its occurrence (22) This might be a common finding in a population suffering from chronic pain, where self-medication can occur (22). Therefore, the TAN could be a more appropriate variable when assessing postoperative pain. This is confirmed in this study, with the TAN being higher in the TG, which correlated with a higher duration and intensity of postoperative pain.

Concerning the individual factors of the patient, patients in the TG had a higher number of general factors, such as pre-surgical pain in the last month (m-VISTA = 41.70% vs. CAF = 33.30%) and in the day of the surgery (m-VISTA = 16.70% vs. CAF = 8.30%) and moderate CLCS (m-VISTA = 16.70% vs. CAF = 0%), systemic diseases (m-VISTA = 50% vs. CAF = 25%), drug intake (m-VISTA = 33.30% vs. CAF = 16.70%), and more women (m-VISTA = 66.70% vs. CAF = 50%). Therefore, the sample in the m-VISTA group may have had a stronger predisposition for postoperative pain.

Both preoperative PI and CLCS were higher in the TG. Although there was a trend towards higher acute PI in patients with a higher CLCS, without statistical significance, probably due to the sample size, which had been calculated for the mean root coverage (main variable) of the previously published RCT (12). Knowledge about pre-surgical CLCS, obtained through the CSI (15), would allow us to identify patients with somatosensory system dysfunctions before surgery (23) and/or the presence of any chronic pain pathologies (24) which could act as a predisposing factor for acute PI or even for developing a chronic pain condition (4,25) This could also serve to assess the suitability of the intervention, as well as the need to modify the surgical technique or to prescribe postoperative medication. Moderate to severe preoperative pain and acute postoperative pain are independent predictors for chronic postoperative pain (25,26). Therefore, we must emphasize the importance of adequate acute postoperative pain control, as it is often associated with increased morbidity, impaired function and quality of life, delayed recovery time, prolonged duration of opioid use, and increased health care costs (27).

It was also noticed that toxic habits (drugs and/or alcohol consumption) could induce a higher postoperative PI. This relationship has not been extensively studied in dentistry, but there is evidence that patients who regularly consume alcohol take more opioids for pain control after surgery (25,28). On the contrary, no association was recognized between smoking and postoperative pain, unlike other authors (25,29) who considered tobacco consumption as a clear indicator (25).

It should be noted that more women were allocated in the TG (66.70%); one of them suffered from fibromyalgia and migraine, and three women had depression and/or anxiety which significantly increased the mean values of the postoperative pain variables recorded, which may partly explain the results obtained in this group. Sex- and gender-related factors have a greater intensity of acute pain and predisposition to chronic orofacial pain. Moreover, it could also be linked to the hormonal complex (4) or to psychological and sociocultural aspects (30).

In view of the above, it could be argued that PI after periodontal surgery might be mainly triggered by the patient's previous conditions. Therefore, studies analyzing postoperative pain should be performed in a homogeneous population, adjusting for other intraoperative and postoperative confounding factors that may increase the pain experience.

This study had some limitations: first, the sample size, which was calculated for the main variable of the previously published RCT (12), which was the mean root coverage in multiple Miller class III/RT2 gingival recessions, so new studies with larger samples are necessary to study the perception of the pain in periodontal surgery. In addition, the fact of using an SCTG in both groups could be a limitation, since it is not possible to differentiate if the pain relates only to the surgical technique (m-VISTA or CAF) in the bed-recipient or to the donor area (palate). However, as it has been previously stated, the patient's perception of pain was a secondary outcome of this RCT, and for the primary outcome (mean root coverage), current evidence considers the use of an SCTG with both tunneling techniques or coronally advanced flaps, as the gold standard, especially in these challenging multiple gingival recessions (Miller class III/RT2). Nevertheless, harvesting the graft from the palate with the same surgical technique in all the cases might have helped to minimize the risk of bias.

Finally, the strengths of this study should also be highlighted. On the one hand, the pre-surgical tool that was implemented in our study (CLCS) could help acknowledge the patient’s profile, as well as assess the risk-benefit of the intervention, and to adapt the postoperative management. All of this might result in an improvement in patient care and the establishment of an individualized healthcare activity. On the other hand, we consider that the use of the "UPV/EHU pain diary" (9) allows us to carry out an adequate analysis of acute pain closest to the intervention, which presents a maximum intensity 48 hours after surgery. However, it must be associated with an adequate recording of the pain-facilitating factors present, such as: systemic diseases, medication, CSCL, pre-surgical pain, other existing pain disorders, altered pain modulation, sleep disorders, toxic habits, psychosocial data, genetic risk factors, therapies, etc.), surgical characteristics (size of the SCTG, surgical time) and postoperative incidences, to eliminate them as confounding factors (10).

Postoperative pain was more intense and lasted longer, with a greater analgesic requirement, in patients who underwent surgery for multiple Miller class III/RT2 recessions using the m-VISTA technique. Nevertheless, these patients had a greater number of risk factors than those in the CG, which may have increased or modified their pain-related experience.

The consolidation of these results requires further clinical studies with larger samples, to eliminate confounding factors in the assessment of pain and to determine the real effect of the surgical technique on its development, so the appropriate preventive and treatment methods can be applied.

## Figures and Tables

**Table 1:** Pre-surgical characteristics of the patients.

Treatment	Patient	Sex	Age	Systemic disease	Medication	Smoking habit			Drugs/alcohol consumption	CLCS	Pre-surgical pain (VAS)	
			(years)			Type	Cig/day	Years			Last month	Surgery day
m-VISTA (n = 12)	1	F	57	Fibromyalgia/ Migraine	Citalopram/ Lorazepam	FS	0	8	No	Mod	60	30
	2	F	42	Hypercholesterolemia/ Arthrosis	No	NS	0	0	No	Subc	50	0
	3	M	57	No	No	NS	0	0	No	Subc	0	0
	4	M	60	Renal insufficiency/ Asthma/ Hypercholesterolemia	Terbutaline/ Rocatrol	FS	0	11	No	Subc	0	0
	5	F	51	No	No	FS	0	32	No	Mod	30	30
	6	F	52	No	No	NS	0	0	No	Subc	0	0
	7	M	62	No	No	NS	0	0	No	Subc	0	0
	8	F	55	Asthma/ Depression	Terbutaline/Budesonide/ Formoterol/Escitalopram	FS	0	11	No	Subc	0	0
	9	F	47	No	No	NS	0	0	No	Subc	0	0
	10	F	57	Anxiety/ Depression	Simvastatin/ Desvenlafaxine	FS	0	16	No	Med	50	0
	11	F	73	Depression	No	NS	0	0	No	Subc	20	0
	12	M	51	No	No	FS	0	16	No	Subc	0	0
CAF (n = 12)	1	F	50	Migraine	No	FS	0	6	No	Med	30	20
	2	F	59	No	No	FS	0	8	Yes	Subc	0	0
	3	M	49	Asthma	Salbutamol	S	10	31	Yes	Subc	0	0
	4	M	51	No	No	S	9	22	No	Subc	0	0
	5	F	58	No	No	FS	0	12	Yes	Subc	0	0
	6	M	56	Arterial hypertension	No	FS	0	27	No	Subc	0	0
	7	F	31	No	Omeprazole	FS	0	6	No	Subc	40	0
	8	M	63	No	No	NS	0	0	No	Subc	0	0
	9	M	31	No	No	S	2	10	Yes	Subc	0	0
	10	F	51	No	No	NS	0	0	No	Subc	30	0
	11	M	59	No	No	NS	0	0	No	Subc	0	0
	12	F	56	No	No	FS	0	36	No	Med	20	0

F: female; M: male; S: smoker; NS: non-smoker; FS: former smoker; CLCS: clinical level of central sensitization; Subc: subclinical; Med: medium; Mod: moderate; VAS: visual analogue scale.

**Table 2:** Surgical and postoperative characteristics of the patients.

Treatment	Patients	SCTG (mm)			Postoperative incidences	Postoperative pain			
		Length	Width	Thickness		PI (VAS)	PD (min)	AI (n)	TAN (min)
m-VISTA (n = 12)	1	25.29	9.69	1.28	No	35.00	56.00	11.00	710.00
	2	38.54	7.00	3.58	NPM	24.77	15.00	1.00	23.00
	3	19.28	7.13	2.38	No	5.45	2.00	0.00	0.00
	4	32.32	8.05	2.72	No	1.82	5.00	0.00	0.00
	5	21.38	5.11	2.03	No	7.41	146.00	1.00	30.00
	6	21.00	10.32	1.98	A	3.18	10.00	0.00	0.00
	7	38.46	7.46	2.70	FH	4.55	1.00	0.00	0.00
	8	40.31	6.20	2.80	A	25.91	29.00	0.00	0.00
	9	32.45	6.04	2.53	NPM	7.50	20.00	0.00	0.00
	10	30.27	8.34	1.32	No	13.18	8.00	0.00	0.00
	11	31.64	6.73	2.27	No	1.82	4.00	2.00	0.00
	12	15.60	7.25	2.74	PB	3.64	6.00	0.00	0.00
	Mean (SD)	28.88 (8.26)	7.44 (1.49)	2.36 (0.65)	Yes (6) / No (6)	11.19 (11.18)	25.27 (41.00)	1.25 (3.13)	63.58 (203.83)
CAF (n = 12)	1	25.31	8.70	3.57	FH	14.68	7.00	5.00	60.00
	2	22.65	7.82	3.76	A	5.45	7.00	0.00	0.00
	3	22.99	5.97	2.55	No	0.00	0.00	5.00	0.00
	4	32.82	6.65	1.89	No	6.82	8.00	3.00	24.00
	5	21.05	10.29	4.15	No	0.00	0.00	0.00	0.00
	6	36.18	6.24	1.75	No	39.82	60.00	0.00	0.00
	7	22.45	9.19	2.19	No	2.27	3.00	1.00	60.00
	8	31.14	7.36	2.94	FH/NPM	1.36	2.00	11.00	35.00
	9	19.41	5.24	2.06	NPM	0.00	0.00	0.00	0.00
	10	24.14	7.53	2.71	PNG	0.00	0.00	0.00	0.00
	11	25.71	3.62	1.69	PNF	25.00	37.00	7.00	420.00
	12	32.30	4.75	2.08	No	1.82	2.00	6.00	40.00
	Mean (SD)	26.35 (5.39)	6.95 (1.93)	2.61 (0.83)	Yes (6) / No (6)	8.10 (12.52)	10.34 (18.67)	3.17 (3.63)	53.25 (117.93)

SCTG: subepithelial connective tissue graft; NPM: necrosis of the palatal mucosa; A: aphthae; FH: facial hematoma; PB: postoperative bleeding; PNG: partial necrosis of the graft; PNF: partial necrosis of the flap; PI: pain intensity; VAS: visual analogue scale; PD: pain duration; AI: analgesic intake; TAN: time of analgesic need.

**Table 3:** Analysis of the potential relationship between postoperative pain intensity (PI) and the other variables recorded.

Variables	Applied test	m-VISTA (n = 12)		CAF (n = 12)		All the patients (n = 24)	
		r	*p value*	r	*p value*	r	*p value*
Age (years)	Spearman's correlation coefficient	-0.434	0.158	0.21	0.512	-0.032	0.883
Presurgical pain in the last month (VAS)		0.513	0.088	-0.013	0.969	0.219	0.305
Presurgical pain in the day of surgery (VAS)		0.389	0.211	0.311	0.325	0.374	0.072
SCTG length		0.326	0.301	0.619*	0.032*	0.418*	0.042*
SCTG width		-0.137	0.672	-0.132	0.683	-0.093	0.666
SCTG thickness		-0.042	0.897	-0.445	0.147	-0.308	0.144
Sex	Mann-Whitney U		0.109		0.699		0.508
Drugs/alcohol			0.073		-		0.023*
Systemic disease			0.639		0.758		0.26
Medication			0.214		0.485		0.673
Presence of presurgical pain in the last month			0.268		0.933		0.599
Presence of presurgical pain in the day of surgery			0.273		0.5		0.082
Postoperative incidences			0.639		>0.05		0.733
Type of smoker	Kruskal-Wallis		0.394		0.499		0.233
CLCS			0.288		0.606		0.259

SCTG: subepitelial connective tissue graft; CLCS: clinical level of central sensitization; *statistically significant value (*p*<0.05).

## Data Availability

The data that support the findings of this study are available from the Correspondence upon reasonable request.
